# Spontaneous resolution of asymptomatic alumina matrix composite ceramic liner dissociation: a case report

**DOI:** 10.1186/s12891-022-05743-6

**Published:** 2022-08-20

**Authors:** He Xiao, Jian Wang, Nian-Ye Zheng, Zhan-Jun Shi

**Affiliations:** 1 Department of Orthopedic Surgery, Nanfang Hospital, Southern Medical University, Guangzhou, China; 2 Department of Orthopedic Surgery, Washington University in St. Louis, 1 Barnes Jewish Hospital Plaza, 63110 St. Louis, MO USA

**Keywords:** Total hip arthroplasty, Hip prostheses, Ceramics, Equipment failure

## Abstract

**Background:**

Total hip arthroplasty(THA)is widely used to treat end-stage hip disorders. Ceramic-on-ceramic total hip prostheses are widely used because of their durability. Alumina matrix composite (AMC), known as the fourth-generation ceramics, reduces implant fracture and wear rate compared to their predecessors. However, ceramic acetabular liner dissociation is a complication that necessitates revision of the AMC prostheses. To date, only few cases of AMC liner dissociation have been reported and all of which have been treated with revision surgery. Therefore, the prognosis of non-operated AMC liner dissociation remains unknown so far.

**Case presentation:**

A 57-year-old man with avascular necrosis of the femoral head was treated with THA, wherein a Pinnacle® (DePuy, J&J, Warsaw, IN) acetabular cup and AMC liner were implanted. Intraoperative examination confirmed proper seating of the liner, whereas the initial postoperative radiograph revealed liner dissociation. The patient refused surgical revision due to the absence of symptoms and was discharged and followed-up. The patient made an uneventful recovery, and radiographic follow-up at 6-month post-operation showed that the liner was re-seated to its right position. No clinical or radiographic anomaly was found at the 15-month of postoperative follow-up.

**Conclusions:**

Here, we report an unprecedented case of AMC ceramic liner dissociation with spontaneous resolution. This case shows that ceramic liner dissociation could be asymptomatic, and careful postoperative examination of the patient is important. Spontaneous resolution is possible, but the underlying mechanism and the eligible patient to benefit from it must be investigated. Before clarifying these questions, revision surgery should be the first-line treatment.

## Background

Since its emergence in the 1970s, ceramic-on-ceramic prostheses for total hip arthroplasty (THA) have undergone continuous improvement, and one of the most often used is the alumina matrix composite (AMC) modular prosthesis [1]. The current acetabular component of ceramic-on-ceramic hip prosthesis is composed of an AMC liner mating intraoperatively with a titanium biological fixation shell through Morse’s taper surface.

The AMC material is popular because of its lower wear rate and lower risk of fracture compared to its predecessors [2]. However, there are still complications for ceramic-on-ceramic hip prostheses, one of which is ceramic liner dissociation. All AMC liner dissociation cases in the literature were immediately rectified with revision due to concerns about adverse outcomes, such as fragmentation [3–6]. To the best of our knowledge, there are no report of dissociation with spontaneous resolution of AMC liner. Here, we report the first case of re-seating of AMC liner.

## Case presentation

**Fig. 1:**
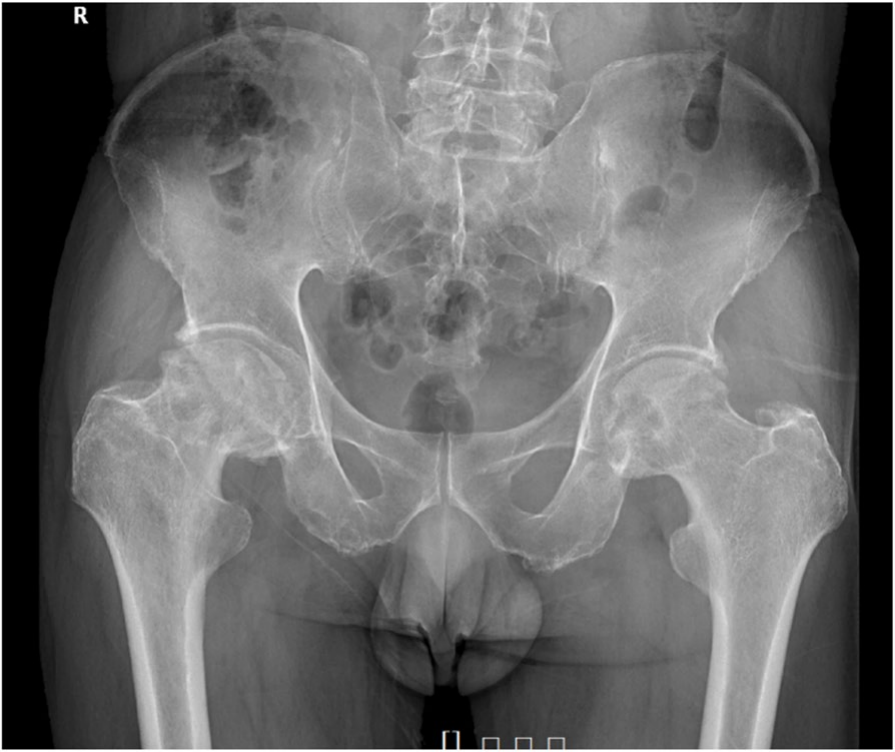
Plain radiograph of pelvis before right hip arthroplasty. Bilateral Ficat IV stage avascular necrosis of femoral head is shown

A 57-year-old man with bilateral primary avascular necrosis of the femoral head, confirmed by postoperative pathology upon radiograph diagnosis , underwent cementless total hip arthroplasty (THA) of the right hip in September 2020 (Fig. 1). The patient weighed 69 kg and had a body mass index (BMI) of 23.3. The operation was performed using Hardinge’s approach, with the patient in a supine position. The implants used were Pinnacle® acetabular with a 54 mm outer diameter, a TRI-LOCK® stem ( Depuy, J&J, Warsaw, IN, USA), a BIOLOX® delta ceramic liner, and a 36 mm head (CeramTec, Plochingen, Germany).

The surgery was conducted routinely by reaming the acetabulum to a 54 mm diameter, implanting a 54 mm acetabular cup, and then seating a 36 mm AMC liner. The cup was implanted with anteversion of 10° and abduction of 40°, and the liner impacted after seating. We ensured proper seating and rigid locking by visual and palpation check and pushing the liner’s rim with a hemostat. The femoral part was completed smoothly. Intraoperative fluoroscopy showed no sign of liner dissociation; however, no images were recorded because the whole procedure was so unremarkable that saving the radiograph seemed not necessary.

**Fig. 2:**
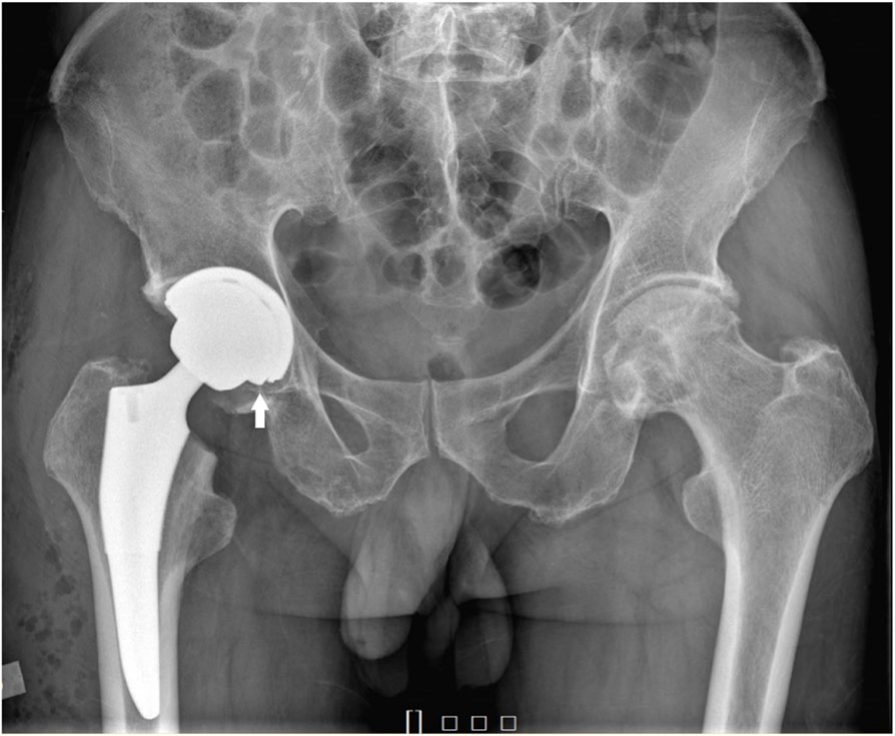
Plain radiograph and computed tomography of pelvis immediately after the surgery. A protruding ceramic liner rim can be observed on a plain hip radiograph the day after THA. (arrow)

On the first postoperative day, a pelvic plain radiograph showed a prominence between the femoral head and the inferior rim of the acetabular cup, which implied dissociation of the ceramic liner (Fig. 2). Considering the consequences of liner fragmentation and other complications, we recommended revision surgery. Although informed of the indication and necessity of revision, the patient refused since he had no symptoms. Given that consent for revision surgery was not forthcoming from the patient, we adopted a non-operative approach with routine rehabilitation protocol same as regular postoperative patients. The option of conducting revision surgery remains available when liner fragmentation or any related discomfort emerged in subsequent follow-up.

**Fig. 3:**
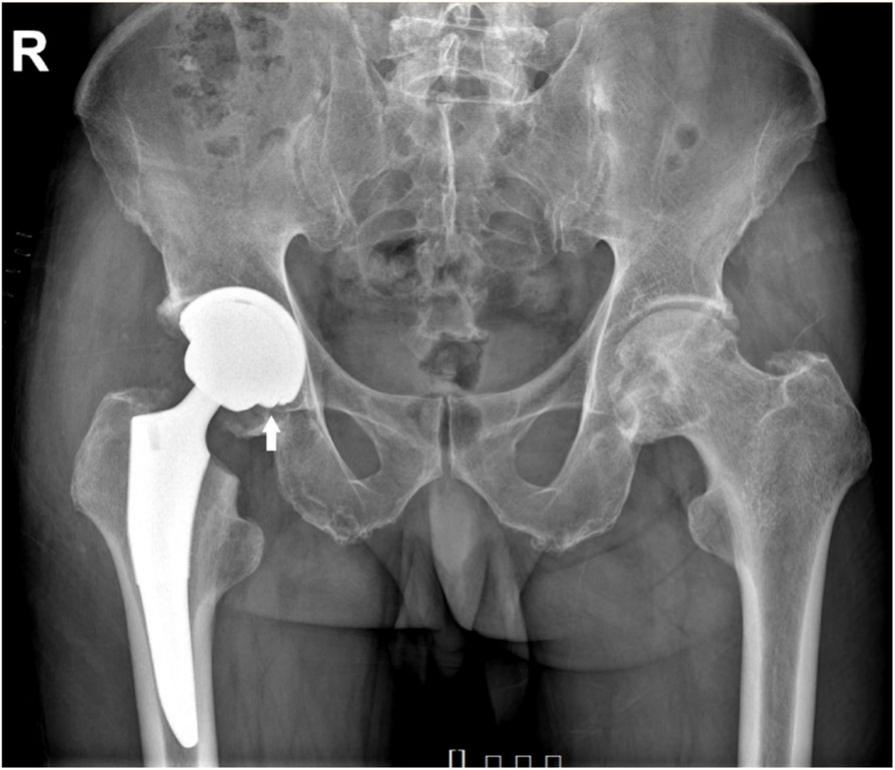
Plain radiograph on the eighth day after the surgery.Ceramic liner dissociation can be observed on a plain hip radiograph (arrow), which is almost identical with the presentation on the radiograph of the first postoperative day (Fig. 2a)

**Fig. 4:**
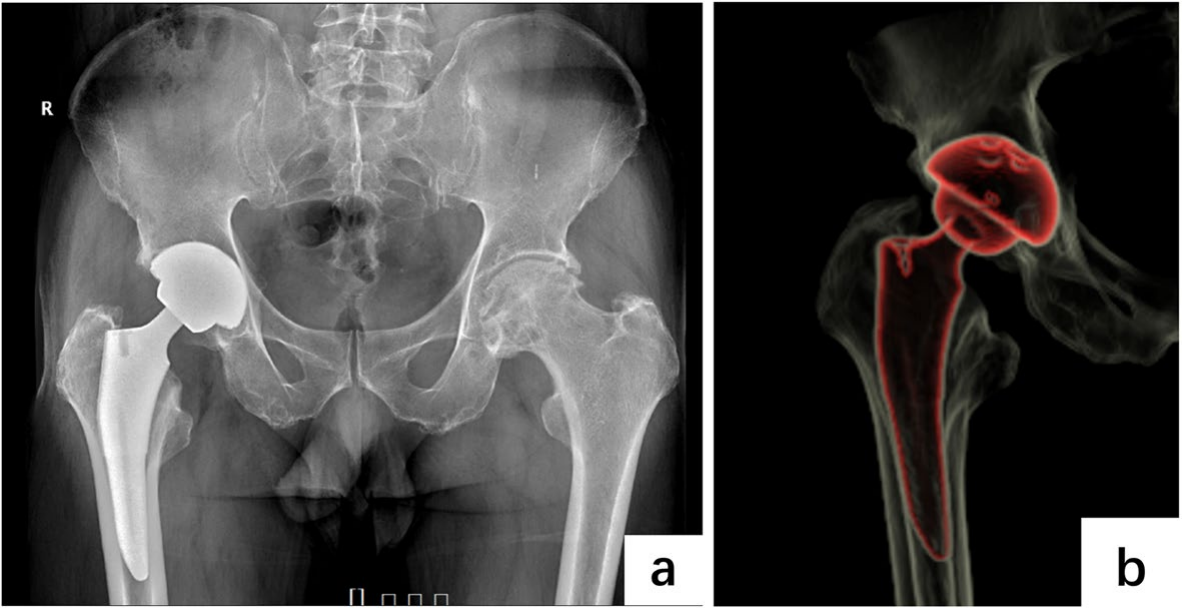
Presentation of spontaneous reduction of the dissociated liner eight months postoperatively.Plain pelvic radiograph shows no protrusion of the ceramic liner. **a** CT contour reconstruction (**b**) confirms proper seating of the liner in the acetabular cup (**b**)

The patient ambulated full weight-bearing on the first postoperative day. On the eighth postoperative day, another radiograph showed no significant change of the liner’s oblique position (Fig. 3). The patient was then discharged, and he returned to most daily activities without assistance six weeks after surgery. Asymptomatic creaking was noticed when the patient rose from a stool eight weeks postoperatively. The rehabilitation was uneventful. Eight months after surgery, the patient was admitted for THA of the contralateral side. His right hip recovered satisfactorily without any pain or functional obstacles during his everyday activities. Surprisingly, the preoperative X-ray demonstrated proper seating of the ceramic liner without prosthesis loosening or migration in the right hip (Fig. 4a), which was further confirmed by a CT scan (Fig. 4b). The oblique right hip acetabular liner spontaneously regained its normal position; hence, no intervention other than observation was prescribed. The surgery on the left side was uneventful. We implanted the same type of prosthesis with appropriate size.

**Fig. 5:**
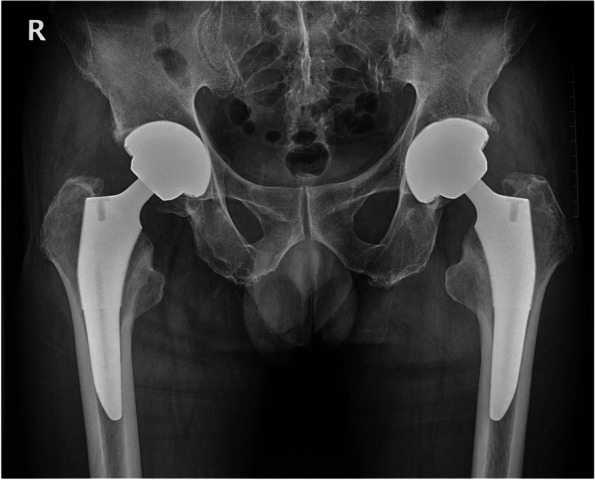
Plain radiograph of pelvis 15 months after right hip arthroplasty. Plain pelvic radiograph shows no protrusion of the ceramic liner. The liner remained properly seated after spontaneous reduction

The patient’s postoperative course was uncomplicated. The latest follow-up of the patient was 15 months from the first THA surgery and 6 months after the discovery of liner spontaneous reduction. He denied any discomfort in the right hip with a Harris hip score of 87, and the radiograph indicated no recurrence of liner dissociation (Fig. 5).

## Discussion and conclusions

There have been few reports of dissociation with AMC prostheses and that all of these have been treated with revision surgery due to concerns of fragmentation. Six AMC ceramic liner dissociation cases in 1103 hips have been reported, all of which were promptly revised on diagnosis [3–6]. In three of these six cases, dissociations were found immediately after surgery, and one just before suturing [3, 6]. The other two AMC liner dissociations were detected after liner fracture, presumed to be a consequence of dissociation [4, 5]. In comparison, 183 cases of liner dissociation have been reported with older liners such as the Trident® (Stryker Orthopaedics, Mahwah, NJ) in 1239 hips [7–13]. Among these cases, with the largest case pool of 694 hips, Miller et al. reported 50 malseating cases (7.2%), among which patients showed good outcomes with non-operative management in the majority of cases, albeit without spontaneous resolution [9]. In addition, the incidence of liner dissociation in direct taper fit 3rd generation ceramic prostheses has been reported to be low (< 0.5%) in three studies on the topic [14–16].

Various hypotheses exist in the literature regarding the etiology of ceramic liner dissociation, including failure to impact the liner with sufficient force [3], interposition of soft tissue or bone fragments [3], deformation of the metal shell [1], excessive rotational torque to the liner owing to wear-roughened joint surfaces [17]. The cause of the dissociation in the present case was unclear. At our center, it is a routine to impact the ceramic liner and femoral head rigorously, and the index surgery was no exception. Also, tissue interposition was carefully excluded intra-operatively. Hence, the properties of the prosthesis might have played a role in liner dissociation in this case. Two design features of the multibearing metal shells have been suggested to increase the risk of dissociation: a relatively short taper surface [18] and a taper angle of 10° rather than 18° [19]. The former was deemed to reduce taper fixation stability, and the latter was shown to hinder correct intraoperative seating of the liner, thus rendering the liners vulnerable to dissociation [19]. However, the number of reported AMC dissociations is very low and it is unknown whether these factors may have contributed to the ceramic liner dissociation reported in this case report.

Ceramic liner dissociation has been considered an indication for revision surgery [20]. And it is rare for dissociated ceramic liners to reseat spontaneously in the proper position without intervention. Only four cases have been reported, all of which were in patients using Trident® prostheses [7, 8]. In ceramic modular liners without a metal or polyethylene sleeve, spontaneous resolution after dissociation has not been recorded. To the best of our knowledge, this is the first case report on the outcome of unrevised direct taper-fit AMC liner dissociation, and the first noted spontaneous resolution of AMC liner dissociation.

The mechanism of spontaneous resolution is unclear, and elastic recoil of the cup might play a role. Langdown et al. reported two cases of spontaneous repositioning of the Trident® ceramic liner after dissociation, proposing that the acetabular shell was deformed during the initial press-fit implantation [7]. Markel et.al’s cadaveric mechanical study showed that the acetabular cup deforms after press-fit implantation but retracts after rest and joint loading [21]. This “settling-in effect” phenomenon supports Langdown’s hypothesis [7] and might explain the present case.

In conclusion, AMC ceramic liner dissociation can be asymptomatic, and careful postoperative examination is important. Spontaneous resolution is possible, but the underlying mechanism and eligible patients who can benefit from it are unknown. Revision surgery should always be considered as the first-line treatment, and extra attention should be paid intra- and post-operatively to procedures involving multibearing metal shells [18, 19]. However, the existence of spontaneous resolution could be helpful for surgeons in the future who may wish to continue with a period of clinical and radiographic observation rather than progressing to early revision.

## Data Availability

The datasets used and/or analyzed during the current study are available from the corresponding author on reasonable request.

## Abbreviations

THA: Total hip arthroplasty
AMC: Alumina matrix composite
HXLPE: Highly cross-linked polyethylene
